# Deformation behavior of Re alloyed Mo thin films on flexible substrates: *In situ* fragmentation analysis supported by first-principles calculations

**DOI:** 10.1038/s41598-017-07825-1

**Published:** 2017-08-07

**Authors:** Tanja Jörg, Denis Music, Filipe Hauser, Megan J. Cordill, Robert Franz, Harald Köstenbauer, Jörg Winkler, Jochen M. Schneider, Christian Mitterer

**Affiliations:** 1 Department of Physical Metallurgy and Materials Testing, Montanuniversität Leoben, Franz-Josef-Strasse 18, 8700 Leoben, Austria; 2 Materials Chemistry, RWTH Aachen University, Kopernikusstrasse 10, 52074 Aachen, Germany; 3 Erich Schmid Institute of Materials Science, Austrian Academy of Sciences, and Department of Materials Physics, Montanuniversität Leoben, Jahnstrasse 12, 8700 Leoben, Austria; 4 Business Unit Coating, PLANSEE SE, Metallwerk-Plansee-Strasse 71, 6600 Reutte, Austria

## Abstract

A major obstacle in the utilization of Mo thin films in flexible electronics is their brittle fracture behavior. Within this study, alloying with Re is explored as a potential strategy to improve the resistance to fracture. The sputter-deposited Mo_1−x_Re_x_ films (with 0 ≤ x ≤ 0.31) were characterized in terms of structural and mechanical properties, residual stresses as well as electrical resistivity. Their deformation behavior was assessed by straining 50 nm thin films on polyimide substrates in uniaxial tension, while monitoring crack initiation and propagation *in situ* by optical microscopy and electrical resistance measurements. A significant toughness enhancement occurs with increasing Re content for all body-centered cubic solid solution films (x ≤ 0.23). However, at higher Re concentrations (x > 0.23) the positive effect of Re is inhibited due to the formation of dual-phase films with the additional close packed A15 Mo_3_Re phase. The mechanisms responsible for the observed toughness behavior are discussed based on experimental observations and electronic structure calculations. Re gives rise to both increased plasticity and bond strengthening in these Mo-Re solid solutions.

## Introduction

Mechanical failure of thin metal films on compliant substrates presents a considerable challenge in the development of flexible electronics. Brittle metal films are often responsible for device failure during deformation^1, 2^. However, these thin films are needed to fulfil the required functionality of the devices and cannot easily be replaced. Mo films are often the primary choice in many electronic applications due to their attractive combination of functional properties. For example, they serve as back electrode materials in flexible CuInGaSe_2_-based solar cells due to their good chemical stability and low contact resistance^3, 4^. They are also used as electrode materials in flexible piezoelectric micro- and nano-electromechanical systems, because of their high acoustic impedance and low electrical resistivity^5, 6^. In addition, Mo films are commonly applied in the metallization of thin film transistors, e.g. gate and source/drain electrodes, as adhesion promotion, diffusion barrier and ohmic contact layers. Thin film transistors are utilized to drive flexible displays, such as active-matrix organic light emitting diode displays^7, 8^. Although Mo films are already used in some flexible systems, their brittle fracture behavior limits the utilization of these devices to low strains or bending radii^3, 9, 10^.

To overcome this problem, strategies have been explored to improve the fracture behavior of Mo films. It is known that alloying with Re improves the room temperature ductility of Mo which results in an increased workability and toughness of the alloys^11–14^. This so-called “Re-effect” is observed for all group VI-A metals (Cr, Mo, W) and has attracted considerable attention in bulk materials^15–17^. The origin of the effect has been debated heavily and several in part contradictive explanations have been suggested. While for small alloy contents (≤8 at.% Re) solid solution softening was proposed^18, 19^, this argumentation fails to explain the increased ductility of high Mo-Re alloys (~30 at.% Re)^12–14^. Others implied that alloying with Re seems to prevent impurity segregation at grain boundaries, although it has been agreed that this cannot be the only reason for the ductility enhancement^20, 21^. Several studies indicated that mechanical twinning plays an important role in the deformation behavior of Mo-Re alloys^12, 13^. While all of these studies concentrated on the mechanical properties of bulk alloys, the aim of this work is to gain insights in the effect of Re on the deformation behavior of Mo films by using a combined approach of experimental techniques and first-principles calculations.

**Figure 1:**
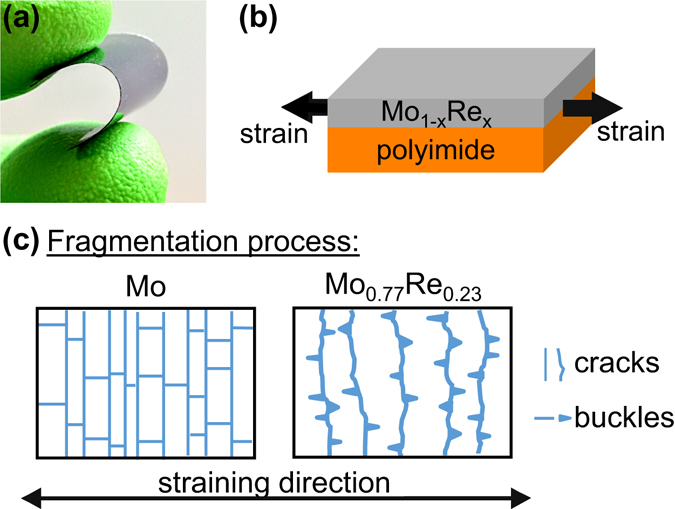
(**a**) Photograph of a sputtered Mo-Re film on flexible PI substrate, schematic representation of (**b**) the uniaxial straining of the film-substrate system and (**c**) the different fragmentation of the Mo and Mo_0.77_Re_0.23_ thin films strained on PI.

In the presented study, Mo-Re thin films with different alloy concentrations are deposited via magnetron sputtering on flexible polyimide (PI) substrates (see Fig. 1(a)). A general analysis of the fracture behavior of the films is conducted by *in situ* fragmentation tests. The experiments are used to evaluate the fracture strain, or crack onset strain (COS), by straining the film-substrate systems in uniaxial tension (Fig. 1(b)), while measuring the change of the films’ electrical resistance. In addition, the fracture process of the films is monitored under the optical microscope to observe crack initiation and growth during deformation (see schematic in Fig. 1(c)). The influence of Re on the fracture behavior of Mo was further investigated by density functional theory (DFT) calculations, which were used to determine the theoretical fracture toughness of Mo-Re alloys and to rationalize the change in electronic structure and bonding upon Re incorporation.

## Results

**Figure 2:**
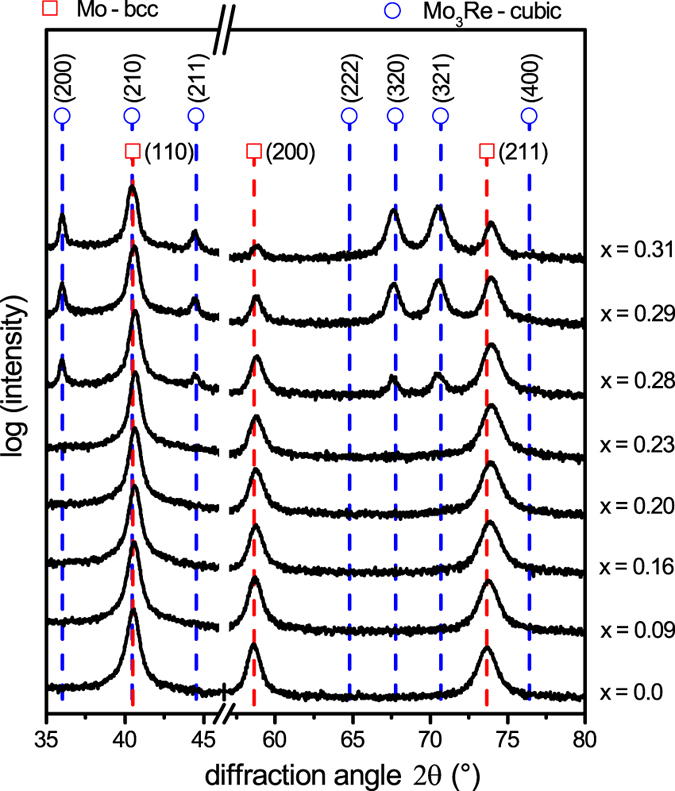
X-ray diffractograms of 500 nm thick Mo_1−x_Re_x_ thin films on Si substrates.

Figure 2 represents the X-ray diffractograms of all deposited Mo_1−x_Re_x_ films for various x values (x in at.% determined by energy dispersive X-ray spectroscopy (EDX)) measured under grazing incidence. The diffraction patterns of the alloyed films corresponding to x ≤ 0.23 indicate formation of a single-phase body-centered cubic (bcc) Mo-based solid solution (space group Im-3m, ICDD 03-065-7442). The patterns of the films with x > 0.23 reveal additional diffraction peaks due to the formation of a second phase. Based on the composition and diffraction data, the peaks can be attributed to the Mo_3_Re compound with an A15 cubic structure (space group Pm-3n, ICDD 01-071-9799).

**Figure 3:**
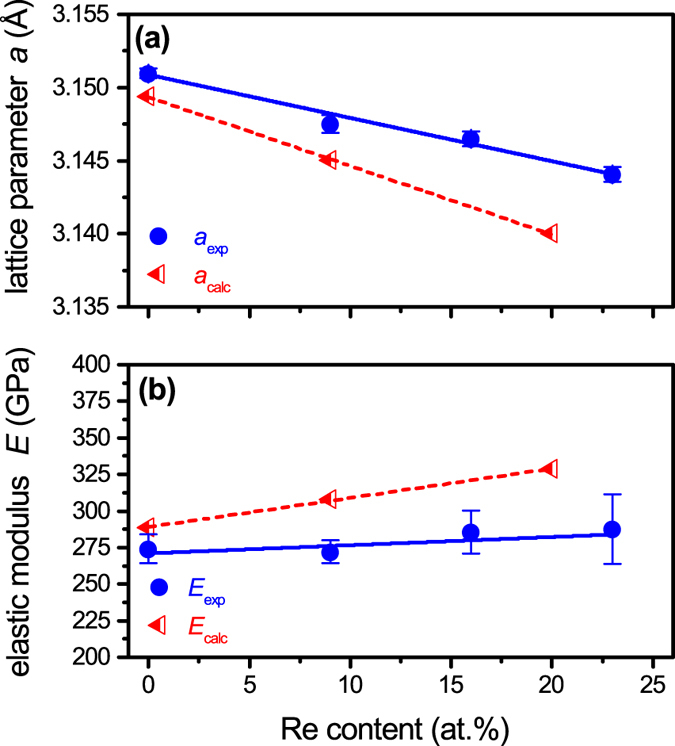
Evolution of the lattice parameter, *a*, and elastic modulus, *E*, as a function of Re content. Full symbols refer to experimental values, while half-open symbols denote calculated values at 0 K.

The evolution of lattice parameter, *a*, and elastic modulus, *E*, for the solid solution regime, determined from both experiments and DFT calculations, is depicted in Fig. 3 as a function of the Re content. For pure Mo, the obtained lattice parameters and elastic moduli are in good agreement with bulk literature values (*a* = 3.147 Å^22^, *E* = 320 GPa^23^). Deambrosis *et al*.^24^ reported a similar value of *E* for sputter-deposited Mo films deviating only 9% from our experimental value. Alloying with Re leads to a linear decrease in *a*, which is associated with the substitution of Mo by smaller Re atoms (atomic radii: r_Mo_ = 1.39 Å, r_Re_ = 1.37 Å^25^). The calculated values of *a* are underestimated by max. 0.2% compared to the experimentally obtained values, both exhibiting the same trend. Figure 3(b) demonstrates that the rising Re content leads to a slightly increasing elastic modulus. The calculated elastic moduli are overestimated by max. 14% with respect to the values obtained by nanoindentation, but the agreement is nonetheless satisfactory. Deviations of the calculated lattice parameters and elastic moduli compared to the experimental values are within acceptable margins based on the employed exchange-correlation functionals^26^.

**Figure 4:**
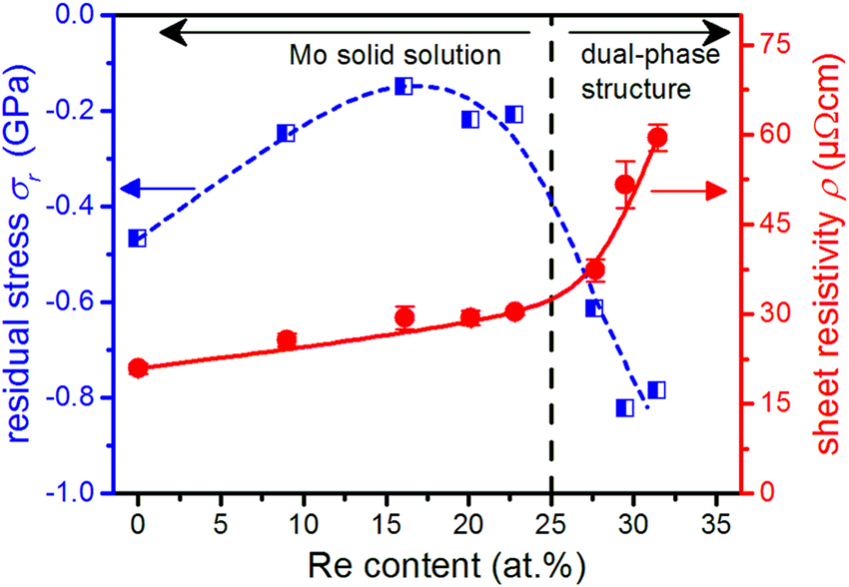
Residual stress, σ_r_, and sheet resistivity, ρ, of 500 nm thick Mo-Re thin films with different Re contents measured on Si substrates. The lines are a guide to the eye and imply no physical meaning.

The evolution of residual stress, σ_r_, and sheet resistivity, ρ, with varying Re content is illustrated in Fig. 4. All deposited films are in a state of compressive residual stress, where the obtained values change with Re content. The compressive stress in the Mo film is likely due to structural defects, which are generated during thin film growth as a consequence of the intense ion bombardment of the film surface at the applied bias voltage^27^. When comparing the pure Mo film with the single-phase solid solution films, a slight relaxation of compressive stress is observed upon Re incorporation. In contrast, the dual-phase films with the Mo_3_Re phase exhibit the highest compressive stress values. Presumably this is related to the formation of the Mo_3_Re compound which exhibits a higher unit cell volume, *V*_*C*_, than Mo (${V}_{{C}_{Mo}}=31.17\,{{\rm{\AA }}}^{3}\,{\rm{ICDD}}\,03-065-7442$, ${V}_{{C}_{Mo3Re}}=123.65\,{{\rm{\AA }}}^{3}\,{\rm{ICDD}}\,01-071-9799$).

Measurements of the electrical sheet resistivity indicate that the electrical properties of the alloyed films strongly depend on the microstructure (see Fig. 4). For the solid solution films, a moderate increase in electrical resistivity with rising Re content is noticed which originates from the higher scattering probability when solutes are induced in the lattice. A more pronounced increase in electrical resistivity is observed for the films consisting of the dual-phase structure. Considering the high resistivity value reported for a single-crystal Mo_3_Re film (98 μΩcm^28^), the rising resistivity indicated an increasing volume fraction of the A15 Mo_3_Re phase. This agrees well to the notion that the resistivity of dual-phase alloys can often be described by rule of mixture behavior^29^.

**Figure 5:**
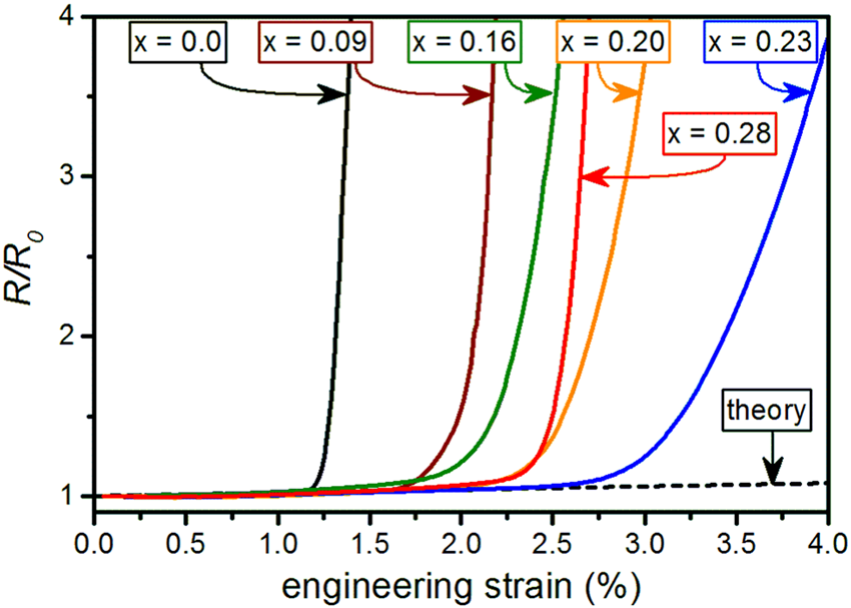
Normalized resistance, *R/R*_*0*_, as a function of the engineering strain of Mo_1−x_Re_x_ thin films with x = 0 as well as 0.09 ≤ x ≤ 0.28. The horizontal dashed line represents the theoretical value based on Eq. (2).

Figure 5 shows the electro-mechanical response of Mo_1−x_Re_x_ films with x ≤ 0.28 during uniaxial tensile tests used to determine the COS of the films. It is evident that the Re content affects the fracture behavior of the films. The pure Mo film remains electrically conductive when subjected to modest elongation (<1% strain). At ~1.1% tensile strain, the normalized resistance starts to deviate from the theoretical line calculated by Eq. (2) due to the formation of cracks. The sharp resistance increase is typical for brittle film fracture^30^. The relative resistance curves of the Mo-Re alloy films illustrate a gradual enhancement in COS with increasing fraction of Re, reaching 2.5% COS for the Mo_0.77_Re_0.23_ thin film. By increasing the Re content above 23 at.%, a reversed trend in the electrical response is noticed (e.g. the film with x = 0.28). The Mo_1−x_Re_x_ films with x > 0.28 are not plotted for better clarity of the graph, however, a progressive decrease in COS was found for those alloyed films.

**Figure 6:**
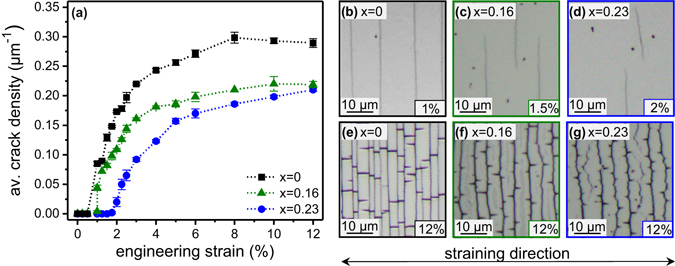
(**a**) Crack density curves of the solid solution Mo_1−x_Re_x_ thin films with x ≤ 0.23 and micrographs of (**b**) Mo, (**c**) Mo_0.84_Re_0.16_, (**d**) Mo_0.77_Re_0.23_ thin films at crack initiation and 12% tensile strain (**e**–**g**).

The beneficial effect of Re on the deformation behavior of the Mo alloy films was further investigated by *in situ* optical fragmentation tests. The evolution of crack density of Mo and two alloyed films (x = 0.16 and x = 0.23) is plotted in Fig. 6(a) over the applied engineering strain. For the Mo film, the first cracks are observed at ~1% tensile strain and travel across the entire sample width perpendicular to the straining direction (see Fig. 6(b)). After the COS is reached, there is a rapid increase in crack density, called mid-point cracking regime after^31^, until a saturation level is reached, where no further cracks can form. A similar trend to the *in situ* resistance measurements is noticed with increasing Re contents. There is a significant shift to higher COS with Re content rising up to x = 0.23. For the Mo_0.84_Re_0.16_ film, crack nucleation occurs at ~1.5% and for Mo_0.77_Re_0.23_ at ~2% tensile strain. The surface micrographs of the Mo_0.84_Re_0.16_ and Mo_0.77_Re_0.23_ films taken at crack initiation (Figs. 6(c–d)) depict that although cracks are present, they have not fully propagated through the whole sample width. As a consequence, the COS values obtained here are slightly lower compared to the *in situ* resistance test results, since the films remain electrically conductive despite the presence of these small cracks. Furthermore, Fig. 6(a) indicates that alloying with Re not only results in a lower crack density, but also the initial slope of the curves is flattened. The influence of Re on the crack pattern is also demonstrated in Figs. 6(e–g), showing the sample surfaces after the maximum tensile strain. Re addition transforms the straight channel cracks detected for Mo to a more deflected, wavy crack path. Delamination in form of buckles, which form parallel to the straining direction between the crack fragments, is observed for all films after exceeding a tensile strain of 10%. However, the shape of the buckles changes with increasing Re content from rectangular (Mo) to triangular (Mo_0.77_Re_0.23_) (see ref. 32 for further details about buckle formation). It should be noted that the dark spots seen in the micrographs originate from oxides, which are formed inevitably during the testing and analyzing process as the films were exposed to ambient atmosphere. As these oxides occur over time and only at the film surface, it is expected that their influence on the deformation behavior is negligible.

**Figure 7:**
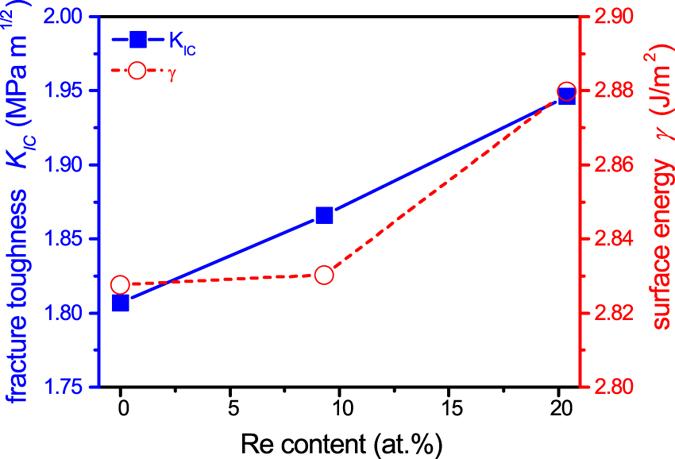
The calculated fracture toughness, *K*_*IC*_, and surface energy, *γ*, for as a function of the Re content.

To rationalize the crack density evolution with varying Re content, the fracture toughness was calculated using DFT. Figure 7 shows the calculated fracture toughness, *K*_*IC*_, and surface energy, *γ*, of Mo_1−x_Re_x_ alloys. The theoretical fracture toughness ${K}_{IC}=\sqrt{4\gamma E}$ is an intrinsic material property, which is related to the surface energy, *γ*, and the elastic modulus, *E*, both obtained from DFT calculations. The surface energy as a function of the alloying content can usually be described with a sinusoidal function, but some deviations may occur due to electronic and strain effects^33^. Here, a slow increase in the surface energy for a low Re content and a larger increment at larger Re contents is observed, which is at least partly due to strain effects (see Fig. 3). The calculated and experimentally determined surface energy of Mo differ by 11%^34^, implying that the calculations are sound. On the other hand, the elastic modulus exhibits a more linear behavior (see Fig. 3). A gradual increase of *K*_*IC*_ is predicted with rising Re content due to the calculated increase in *γ* and *E*. This trend is consistent with the crack density (Fig. 6) and sheet resistivity (Fig. 4) increase upon Re incorporation within the bcc solid solution.

**Figure 8:**
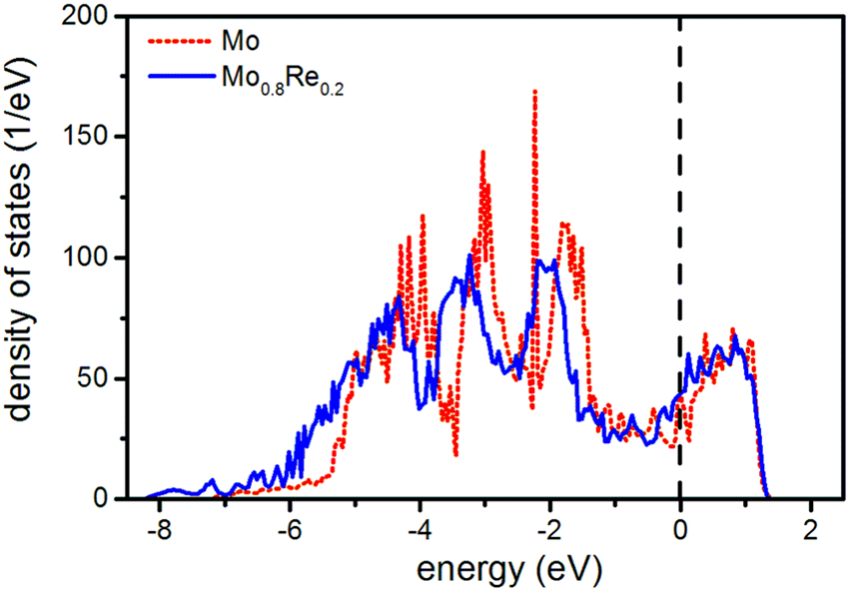
Total DOS for Mo and Mo_0.8_Re_0.2_, as obtained by the VASP code. The Fermi level is aligned with 0 eV and depicted with a vertical dashed line.

To identify a possible physical origin of the fracture toughness enhancement upon Re incorporation, the electronic structure of these solid solutions was studied. The electronic density of states (DOS) data are presented in Fig. 8 for pure Mo and for the Mo_0.8_Re_0.2_ solid solution, where the vertical dashed line corresponds to the Fermi level. As the Fermi level is populated, electrical conductivity is expected, which is consistent with the sheet resistivity data obtained (Fig. 4). The electronic states in these configurations can be characterized by delocalization and hybridization, which is consistent with metallic and covalent interactions, respectively. Both Mo d and Re d states dominate the electronic structure in the vicinity of the Fermi level, while their s and p states become relevant below −6 eV and are thus less significant for the overall bonding and macroscopic behavior. As there are Mo d – Mo d, Mo d – Re d, and partly Re d – Re d overlaps in the DOS data, these orbitals give rise to hybridization. Ravindran and Asokamani have investigated the phase stability of many intermetallics, including Ti-Al, Ti-Fe, and Zr-Al, and correlated their phase stability with an electronic structure fingerprint^35^. They have suggested that a peak in the vicinity of the Fermi level infers electronic structure perturbations, implying a reduced phase stability. Hence, a compound is very stable if the Fermi level is positioned in a pseudogap, separating the bonding and antibonding states^35^. In the case of Mo-Re solid solutions, the d-d interactions give rise to such a pseudogap. Upon Re incorporation, all states shift to lower energies, which implies that Re most likely decreases the population of the antibonding states and hence stabilizes the solid solutions. It should be stressed that this is an unexpected effect as Re (5d^5^ 6 s^2^) possesses an additional valence state compared to Mo (4d^5^ 5s^1^). Furthermore, it can be speculated that this effect is reversed at higher Re contents once the solubility limit has been crossed (see Fig. 2), but this is beyond the scope of this DFT investigation. There is another important consequence of this electronic structure modulation. Shifting hybridized states to lower energies implies stronger bonding^36, 37^. Hence, the observed increase in both, the surface energy and elastic modulus, can be related to this bond strengthening effect, which in turn leads to increasing fracture toughness upon Re incorporation.

## Discussion

The present findings exemplify the significant influence of Re on the fracture behavior of Mo thin films. The COS obtained from fragmentation testing is used as a means of providing toughness estimations for thin films on compliant substrates. The higher the COS, the higher is the film’s resistance to cracking under uniaxial tensile loading. While the pure Mo film exhibits the lowest COS, a remarkable increase in COS can be achieved by Re incorporation. However, this effect is only observed for Mo-Re films consisting of a single-phase solid solution. Beyond the solid solution regime (>23 at.% Re), a dual-phase region emerges, composed of the solid solution and the non-equilibrium Mo_3_Re phase^38^. The appearance of this close packed A15 Mo_3_Re phase correlates with the noted deterioration of the electro-mechanical response and decrease in COS (see x = 0.28 in Fig. 5). Gavaler *et al*. were the first to report on this A15 phase in sputter-deposited Mo-Re thin films^39^. The Mo_3_Re phase was subsequently studied by several authors because of its superconductive properties below ~15 K^28, 40^. Thus, the beneficial effect of Re on the fracture behavior of sputter-deposited Mo films is limited by the solubility of Re in Mo.

When comparing COS values, it is important to consider the films’ residual stress state^41, 42^. We demonstrated in a previous publication^9^ that COS can be significantly increased by inducing compressive residual stress in the film. However, Fig. 4 shows that the compressive stress values of the solid solution films are reduced with increasing Re content, confirming that the increase in COS with rising Re content is essentially an alloying effect and not caused by increasing compressive stress levels within the films.

In order to understand the origin of such a toughness enhancement, the electronic structure of Mo and the Mo_0.8_Re_0.2_ alloy was investigated, indicating that all bonds are strengthened upon Re incorporation. Usually with an increased bond strength an increase in elastic modulus is expected, while the interatomic distance (i.e. the lattice parameter) should decrease^43^. The observed trend in the lattice constant and the elastic modulus of the Mo-Re alloys is in agreement with this general tendency (see Fig. 3). Fracture toughness calculations revealed that Re incorporation beneficially affects the fracture behavior of Mo, yielding an 8% increase in *K*_*IC*_ for x = 20 at.% (see Fig. 7). According to Griffith’s theory, the elastic energy required to cause fracture through crack propagation is balanced by the energy required to create new crack surfaces by bond breaking^44, 45^. Thus, the surface energy calculated by DFT is related to the energy necessary to break atomic bonds^46^. As a consequence, the increase in *K*_*IC*_ with increasing Re concentration can be attributed to the change in electronic structure and the increasing bond strength.

While the calculations of the theoretical fracture toughness only consider elastic deformation, the fragmentation experiments evidence that plastic deformation plays an important role in the fracture behavior of Mo-Re films. If a material is able to deform plastically, it can absorb more energy before fracture occurs. The plastic deformation reduces the stress concentration at the crack tip, blunts the crack and consequently slows down the crack growth^45^. Figures 6(b–d) clearly demonstrate that the crack propagation is retarded in the Mo_0.77_Re_0.23_ film, since the nucleated cracks are not able to propagate immediately across the whole sample width, as it is the case for the Mo film. Furthermore, the micrographs demonstrate a distinctive change in the deformation behavior. The pure Mo film displays a brittle cleavage fracture behavior with straight channel cracks (Fig. 6(e)). With increasing Re content, the crack path becomes more deflected leading to wavy, zig-zag cracks (Fig. 6(g)), which indicates an enhanced ability for plastic deformation. Gruber *et al*.^47^ reported a similar change in crack patterns as the contribution of plastic deformation increases in Ta/Cu thin films on polyimide.

It is assumed that the gain in plasticity in the Mo-Re films is due to an increased activation of deformation twinning. Jaffee *et al*.^12^ investigated the low-temperature tensile properties of Mo-Re bulk alloys. They reported an enhanced tensile strength and ductility upon Re incorporation and observed an increased amount of deformation twins in the microstructure of the deformed alloys. Similar results were also obtained by Agnew and Leonhardt^13^. The increased tendency to deformation twinning has been shown to be caused by a reduction of stacking fault energy in the {112} plane as well as by a decreasing shear resistance of the lattice when alloying Re to Mo^48^.

## Conclusions

*In situ* fragmentation tests were carried out to investigate how Re alloying affects the fracture behavior of Mo thin films. The results reveal a significant increase in crack onset strain (fracture strain) with increasing Re content up to the solubility limit. While the pure Mo thin film exhibits the lowest resistance to fracture upon tensile straining, the Mo-Re films are able to withstand higher tensile strains before failing. However, once the solubility limit is reached (>23 at.% Re), the appearance of the close packed A15 Mo_3_Re phase deteriorates the beneficial effect leading to a decrease in crack onset strain. The observed enhancement in the fracture behavior of the solid solution Mo-Re thin films was assessed theoretically by calculations of the fracture toughness which increases linearly upon Re incorporation. Density functional theory calculations of the electronic structure indicated bond strengthening upon Re incorporation, explaining the improving fracture toughness as both surface energy and elastic modulus increase concomitantly. While the bond strengthening effect is partly responsible for the overall toughness enhancement, experimental findings indicate that another important contribution stems from the increased plasticity of the Mo-Re thin films.

## Methods

### Thin film deposition

The films were synthesized using a lab-scale direct current (dc) magnetron sputter system, equipped with powder metallurgical targets of Mo and Re (∅ 50.8 mm, powder purities of 99.97% and 99.85%, provided by PLANSEE SE), which were mounted on two unbalanced AJA A320-XP magnetrons. The substrate materials, 50 μm thick PI sheets (UBE UPILEX-S 50 S) and 350 μm thick Si (100), were fixed with Kapton tape on the rotatable sample holder opposite to the targets with a target-to-substrate distance of 40 mm. After a base pressure of less than 10^−3^ Pa was reached, plasma etching of the substrates was performed by applying an pulsed dc discharge of −290 V and 50 kHz and an Ar gas pressure of 1 Pa for 2 min. For film depositions, the Ar working gas pressure was reduced to 0.38 Pa and a pulsed bias potential of −50 V was applied to the substrates, while no intentional substrate heating was used. Different Re contents within the films were achieved by altering the dc current on the Re target between 0 and 0.2 A, whereas the current applied to the Mo target was held constant at 0.35 A. The deposition time was varied between 12 and 17 min to obtain 500 nm thick films grown on Si substrates used for microstructural characterization. In a second deposition run, the deposition time was reduced to the range from 1 to 1.5 min to synthesize films on PI substrates with thickness of ~50 nm, while all other parameters remained constant.

### Thin film characterization

The film thickness was evaluated by an optical 3D white light profiling system (Veeco Wyko NT 1000). The chemical composition of the films on Si substrate was analyzed with an EDX detector (Oxford Instruments INCA) attached to the scanning electron microscope (Zeiss Evo-50). The crystallographic structure was investigated by X-ray diffraction in θ/2θ and grazing incidence (2° incidence angle) geometry utilizing a Bruker-AXS D8 Advance diffractometer equipped with Cu-K_α_ radiation and parallel beam optics. The lattice parameter was determined from the θ/2θ measurements by Rietveld refinement^49^ using the fundamental parameter approach^50^ contained within the software TOPAS V4 (Bruker AXS).

Nanoindentation on the 500 nm thin films on Si was performed in a Hysitron TI 950 TriboIndenter equipped with a Berkovich diamond tip. The area function of the tip was determined before and after the experiments using a fused silica sample, yielding the same result. On each sample, 25 load-controlled quasi-static indents were carried out. To cover a suitable range of indentation depths, the indentation load was incrementally decreased with each indent, from a maximum load of 1.5 mN to a minimum of 0.5 mN. The elastic modulus was obtained from the unloading segment applying the Oliver and Pharr method^51^ using elastic modulus, *E*, and Poisson’s ratio, *ν*, of the diamond indenter (*E* = 1141 GPa and ν = 0.07). At least 18 indents on each film with a maximum indentation depth of 10% of the film thickness were performed to achieve reasonable statistics.

The macroscopic biaxial residual stress, σ_r_, of the films on Si substrates was determined using the curvature method applying the modified Stoney’s equation^52^

1 $${\sigma }_{r}={{\rm{M}}}_{s}\frac{{t}_{s}^{2}}{6t}\frac{1}{r},$$

where *M*_*s*_ is the biaxial modulus of the (100) oriented Si substrate (*M*_*s*_ = 180 GPa^53^), *t*_*s*_ and *t* are the substrate and film thickness, and *r* the radius of curvature. The curvature of the substrate was measured with a custom-built device utilizing the reflection of two parallel laser beams. A Jandel RM2 four-point probe was used to evaluate the influence of alloying with Re on the electrical sheet resistivity of the films.

The fracture process of the 50 nm thin films on PI under uniaxial tensile load was monitored *in situ* by measuring the change in electrical resistance, which is a useful technique to evaluate the critical COS. For each film-substrate system, three samples (5 mm × 35 mm) were strained with an MTS Tyron 250 universal testing machine. The electrical resistance during loading and unloading was determined by four-point probes which were incorporated into the grips of the tensile stage as described in ref. 54. The samples were loaded continuously to a maximum elongation of 15% with an initial gauge length and displacement rate of 20 mm and 5 μm/s, respectively. The failure strain (COS) was defined as the strain at which the measured resistance deviates from the theoretical resistance ratio

2 $$\frac{R}{{R}_{0}}={(\frac{L}{{L}_{0}})}^{2}\equiv {(1+\varepsilon )}^{2},$$

where *R*/*R*_0_ is the relative resistance and *L*/*L*_0_ the relative elongation. The engineering strain, *ε*, is defined as $\varepsilon =(L-{L}_{0})/{L}_{0}$. Thus, Eq. (2) was employed to calculate the theoretical resistance ratio for each engineering strain, which is valid as long as no structural changes arise during the experiment and volume conservation is satisfied. However, once the formation of cracks occurs, the resistance ratio of the films cannot longer be describe by an analytical formula^54, 55^.

*In situ* optical fragmentation tests were conducted in order to observe crack initiation and growth during straining as well as to assess the COS independently from the electrical measurements. The experiments were carried out by mounting an Anton Paar TS600 straining device under an Olympus BX51 optical microscope. The samples (7 mm × 35 mm) were strained with a loading rate of 10 μm/s in small increments until the maximum tensile strain of 12% was reached. During straining, surface images were taken which were analyzed with the software Image J^56^ to obtain the crack density at each strain. In every micrograph three lines were plotted perpendicularly to the direction of the cracks and the number of cracks intersecting with the lines was counted. The average crack density was calculated as ratio between the average number of cracks and the length of the lines.

### Computational details

First-principles calculations based on DFT were employed to predict the elastic modulus and fracture toughness of bcc Mo-Re solid solutions^57^. The DFT study was carried out using the Vienna *ab initio* simulation package (VASP) and projector augmented wave potentials^58, 59^ within the generalized-gradient approximation parametrized by Perdew, Burke and Ernzerhof^60^. The Blöchl corrections were applied for the total energy^61^ and the integration in the Brillouin zone was carried out on Monkhorst-Pack^62^ 7 × 7 × 7 k-points. Full structural optimization was carried out for every solid solution within the convergence criterion for the total energy of 0.01 meV and a 500 eV cut-off. Special quasirandom structures^63^, as implemented in the locally self-consistent Green’s function software package^64^, were employed to describe random Mo-Re solid solutions (3 × 3 × 3 supercells, 54 atoms). The Warren-Cowley short range order parameter^65^ within six coordination shells was used to account for randomness. By fitting the total energy-volume data to the Birch-Murnagham equation of state^66^, equilibrium volume and bulk modulus for each configuration were obtained. All elastic constants (*C*_*11*_, *C*_*12*_, *C*_*44*_) were calculated by structural distortion and fitting the energy-distortion data with a second-order polynomial function^67^. The elastic modulus and Poisson’s ratio were obtained from the elastic constants within the Hill approximation^68^. The fracture toughness under tensile loading was calculated from the elastic modulus and surface energy data. The surface energy was obtained within the stoichiometric slab model. More details on the calculation of surface energy^69^ and fracture toughness^70^ can be found elsewhere. The electronic structure was explored by calculating DOS centered at the gamma point in the reciprocal space.

### Data Availability

The datasets generated during the current study are available from the corresponding author on reasonable request.
